# Proteomic profiling of human plasma and intervertebral disc tissue reveals matrisomal, but not plasma, biomarkers of disc degeneration

**DOI:** 10.1186/s13075-025-03489-9

**Published:** 2025-02-10

**Authors:** Christabel Thembela Dube, Hamish T. J. Gilbert, Niamh Rabbitte, Pauline Baird, Sonal Patel, Jeremy A. Herrera, Ivona Baricevic-Jones, Richard D. Unwin, Danny Chan, Kanna Gnanalingham, Judith A. Hoyland, Stephen M. Richardson

**Affiliations:** 1 Division of Cell Matrix Biology and Regenerative Medicine, School of Biological Sciences, Faculty of Biology, Medicine and Health, The University of Manchester, Manchester, M13 9PT UK; 2 Manchester Cell-Matrix Centre, Division of Cell Matrix Biology and Regenerative Medicine, School of Biological Sciences, Faculty of Biology, Medicine and Health, The University of Manchester, Oxford Road, Manchester, M13 9PT UK; 3 Guy Hilton Research Centre, School of Life Sciences, Keele University, Stoke-on-Trent, ST4 7QB UK; 4 Stoller Biomarker Discovery Centre, Faculty of Biology, Medicine and Health, The University of Manchester, Manchester, M13 9PT UK; 5 Division of Cancer Sciences, School of Medical Sciences, Faculty of Biology, Medicine and Health, The University of Manchester, Manchester, M13 9PT UK; 6 School of Biomedical Sciences, Faculty of Medicine Building, The University of Hong Kong, 21 Sassoon Road, Pokfulam, Hong Kong SAR China; 7 Department of Neurosurgery, Manchester Academy of Health Science Centre, Salford Royal Hospital, Northern Care Alliance NHS Foundation Trust, Stott Lane, Salford, M6 8HD UK

**Keywords:** Intervertebral disc, Degeneration, Histological grading, Extracellular matrix, Proteomics, Biomarkers, Plasma

## Abstract

**Background:**

Intervertebral disc (IVD) degeneration is a common cause of low back pain, and the most symptomatic patients with neural compression need surgical intervention to relieve symptoms. Current techniques used to diagnose IVD degeneration, such as magnetic resonance imaging (MRI), do not detect changes in the tissue extracellular matrix (ECM) as degeneration progresses. Improved techniques, such as a combination of tissue and blood biomarkers, are needed to monitor the progression of IVD degeneration for more effective treatment plans.

**Methods:**

To identify tissue and blood biomarkers associated with degeneration progression, we histologically graded 35 adult human degenerate IVD tissues and matched plasma from the individuals into two groups: mild degenerate and severe degenerate. Mass spectrometry was utilised to characterise proteomic differences in tissue and plasma between the two groups. Top differentially distributed proteins were further validated using immunohistochemistry and qRT-PCR. Additionally, correlational analyses were conducted to define similarities and differences between tissue and plasma protein changes in individuals with mild and severe IVD degeneration.

**Results:**

Our data revealed that the abundance of 31 proteins was significantly increased in severe degenerated IVD tissues compared to mild. Functional analyses showed that more than 40% of these proteins were matrisome-related, indicating differences in ECM protein composition between severe and mild degenerate IVD tissues. We confirmed adipocyte enhancer-binding protein 1 (AEBP1) as one of the most significantly enriched core matrisome genes and proteins as degeneration progressed. Compared to others, AEBP1 protein levels best distinguished between mild and severe degenerated IVD tissues with an area under the curve score of 0.768 (95% CI: 0.60–0.93). However, we found that protein changes from associated plasma exhibited a weak relationship with histological grading and AEBP1 tissue levels. Given that systemic plasma changes are complex, a larger sample cohort may be required to identify patterns in blood relating to IVD degeneration progression.

**Conclusions:**

In this study, we have identified AEBP1 as a tissue marker for monitoring the severity of disc degeneration in humans. Further work to link alterations in tissue AEBP1 levels to changes in blood-related proteins will be beneficial for detailed monitoring of IVD degeneration thereby enabling more personalised treatment approaches.

**Supplementary Information:**

The online version contains supplementary material available at 10.1186/s13075-025-03489-9.

## Background

The intervertebral disc (IVD) is a fibrocartilaginous tissue that sits in-between adjacent vertebrae and is comprised of a central gel-like nucleus pulposus (NP) enclosed within an outer thick ring of annulus fibrosus (AF). The IVD contains a heterogeneous, integrated network of extracellular matrix (ECM) and cells that support IVD structure and function, thereby providing mechanical support and flexibility to the spine [1]. Over time, the IVD’s structure and function gradually deteriorate due to ageing, injury, or repetitive mechanical stress. IVD degeneration results in loss of NP hydration, ECM degradation, and neovascularisation and neoinnervation, (which ultimately extends through the AF into the NP). Altogether, these pathophysiological changes result in loss of disc height, bulging and often compression of nerve roots, which contributes to the pathogenesis of low back pain (LBP), a debilitating condition affecting millions of individuals globally each year [2, 3].

IVD degeneration is primarily diagnosed through a combination of physical examinations and imaging techniques. Magnetic resonance imaging (MRI) is the most commonly used technique to visualise and monitor the extent of degeneration through identification of gross features such as loss of disc height, bulging and loss of water content [4]. However, MRI techniques only provide a macroscopic view of the IVD and lack detail of changes to the microscopic and molecular structure of the tissue such as alterations in cell density and ECM that are observed in degenerated IVD histologically [5]. This means that small changes to the structure of the tissue as degeneration worsens may not be detected through MRI scans, and as such imaging techniques have limited capacity to detect or monitor ongoing degenerative changes. Thus, there is a need for improved techniques to monitor progression of IVD degeneration to enable tailored and more effective treatment plans.

A combination of tissue and blood biomarkers may offer a better alternative for monitoring degenerative changes over time. For example, in cancer studies, the use of combined serum or plasma and tissue biomarkers was shown to improve early detection as well as the sensitivity and specificity of diagnostic tests [6, 7]. However, identifying serum/plasma and tissue biomarkers for IVD degeneration has remained challenging due to the heterogeneous nature of the disease [8]. Past studies have demonstrated changes in inflammatory proteins, such as IL-6, CCL5 and TNFA, in serum from patients with degenerated IVD tissue compared to non-degenerated IVD tissue [9–11]. Similar changes in CCL5 and TNFA levels were reported in degenerate IVD tissue, at the gene expression level, in degenerated IVD in comparison to non-degenerate IVD [12, 13]. However, these studies were independent and there have been no attempts to stratify changes seen in the IVD tissue during degeneration with those observed in serum or plasma [14]. Furthermore, with more focus given to comparing non-degenerate and degenerate IVD tissues, limited work has been undertaken to understand whether alterations in IVD tissue protein composition, particularly the ECM, are exacerbated as degeneration worsens and how these changes may affect or reflect blood protein composition.

The IVD’s ECM comprises a complex network of matrisome and matrisome-associated components that interact to provide mechanical support. The central NP is mainly composed of proteoglycans, primarily aggrecan, that maintain hydration and osmotic pressure, which helps the disc resist compressive forces while maintaining flexibility. The AF predominantly consists of collagens, primarily type I, that provide tensile strength and resistance to shear forces thereby maintaining the disc’s structural integrity. IVD cells constantly remodel their local ECM environment, maintaining a balance between synthesis and degradation, a process driven by proteases including matrix metalloproteinases (MMPs) and a disintegrin and metalloproteinase with thrombospondin motifs (ADAMTSs) [15, 16]. During degeneration, this balance is disrupted resulting in increased ECM degradation and functional impairment. IVD degeneration is irreversible and worsens over time; however, ECM changes that drive degeneration progression have not been fully elucidated. In this study, we characterised changes in ECM protein composition of tissues from patients with mild and severe IVD degeneration to understand how degeneration progression affects the ECM environment in the disc. We also examined the protein composition of matched plasma samples to identify any changes that correlated with those observed in the severe and mild degenerated IVD tissues. We propose that a combination of specific tissue and plasma biomarkers is essential for early diagnosis, monitoring, and personalisation of the treatment for IVD degeneration.

## Methods

### Human intervertebral disc tissues

Human IVD tissues were obtained from individuals undergoing lumbar discectomy surgery to treat degenerative disc disease. Full written informed consent was provided by all donors before tissue collection. This study was reviewed and approved by the National Research Ethics Service (17/LO/1408). All experiments were conducted in compliance with the committee’s ethical standards and guidelines. IVD tissues were collected from 18 males and 17 females with a mean age of 41.3 ± 10.4 years (Table 1). Excised tissues were stored in high-glucose Dulbecco’s modified eagle medium (DMEM, *Sigma Aldrich*) following surgery and processed the same day. Whole blood was also collected from these donors to obtain plasma.

### Tissue processing and histology

IVD tissue samples were fixed in 10% neutral buffered formalin (*Sigma-Aldrich*) for 20–24 h and embedded in paraffin blocks. Sections (5 μm) were cut, placed on glass slides, deparaffinised through xylene, and rehydrated in decreasing concentrations of ethanol. Sections were stained with Mayer’s haematoxylin (*Solmedia Laboratory Suppliers*) for 2 min and washed with running tap water for 5 min. Sections were then counterstained with an eosin-Y alcoholic solution with phloxine (*Sigma-Aldrich*) for 10 s, dehydrated, cleared, and then mounted with coverslips. IVD tissues were histologically graded (ranging from 0 to 12) by an experienced histopathologist using the published scoring system described by Sive et al. [5]. The 35 IVD tissues used in this study were classified into mild degenerate (Grades 4–7, *n* = 18) and severe degenerate (Grades 10–12, *n* = 17) (Table 1). No tissues were classed as non-degenerate (Grades 0–3) as all samples were obtained from individuals undergoing treatment for disc degenerative disease diagnosed through MRI imaging.

### Tissue mass spectrometry: protein extraction and liquid chromatography-tandem mass spectrometry (LC-MS-MS)

To extract protein for mass spectrometry, 25 μl of solubilisation buffer (5% (w/v) SDS in 50mM triethylammonium bicarbonate (TEAB), pH 7.55) was added to deparaffinised, rehydrated IVD tissue sections (2 × 5 μm per tube) and incubated at 95 °C for 20 min followed by 60 °C for 2 h in a thermomixer set to 1400RPM. After cooling to room temperature (RT), 75 μl of 5% (w/v) SDS, 10 M Urea, 50mM TEAB, and 13.33mM DTT (pH 7.55) were added to the samples. All samples were transferred to Covaris tubes and sonicated using a focused ultrasonicator (LE220-plus, Covaris) at 8 W for 20 min (sonicated for 300 s, peak power = 180, average power = 72, duty factor 40%, cycles per burst = 200, delay 15s, then repeated once). Reduced disulfide bridges were alkylated by adding 8 μl of 20mM iodoacetamide and incubating at RT in the dark for 30 min. Lysates were acidified with 12 μl of 12% (v/v) phosphoric acid. Acidified lysates were then centrifuged at 12000xg for 5 min and the supernatant was collected into a clean tube prior to proteolytic digestions using suspension trapping (S-Trap). 600 μl of S-Trap binding buffer (90% (v/v) methanol, 100 mm TEAB, pH 7.1) was added to each lysate, and the total volume was transferred to a micro S-Trap spin column (*Profiti*). Samples were washed ten times with 150 μl S-Trap binding buffer. After washing, 2 μg of trypsin (*Promega*) diluted in 25 μl TEAB (pH 8) was added to the S-Trap and columns were incubated at 47 °C for 1 h. Digested peptides were eluted by the addition of 40 μl 50mM TEAB (pH 8) followed by 40 μl of 0.2% (v/v) formic acid. Hydrophobic peptides were eluted by 40 μl of 30% (v/v) acetonitrile, 0.2% (v/v) formic acid and the total volume (120 μl) was collected and lyophilised in a SpeedVac.

Peptides were resuspended in 100 μl of 3% (v/v) acetonitrile, 0.1% (v/v) formic acid and desalted using POROS Oligo R3 beads (*Thermo Fisher Scientific*) in 0.2 μm polyvinylidene fluoride filter plates (*Corning*). Briefly, peptides were mixed with R3 beads for 5 min at 800RPM using a thermomixer. Samples were then washed 10 times with 100 μl 0.1% formic acid for 2 min. Peptides were eluted in 50 μl of 30% acetonitrile. Elution was repeated to obtain a total volume of 100 μl. Samples were lyophilised and stored at 4 °C. For liquid chromatography with tandem mass spectrometry (LC/MS/MS), peptides were resuspended in 10 μl of 5% (v/v) acetonitrile, 0.1% (v/v) formic acid and analysed using an UltiMate 3000 RSLC (*Dionex Corporation*) coupled to a Q Exactive HF Orbitrap (*Thermo Fisher Scientific*) mass spectrometer. Peptide mixtures were separated using a multistep gradient from 95% A (0.1% (v/v) formic acid in water) and 5% B (0.1% (v/v) formic acid in acetonitrile) to 7% B at 1 min, 18% B at 58 min, 27% B in 72 min and 60% B at 74 min at 300nL/min, using a 75 mm × 250 μm inner diameter 1.7 μM CSH C18, analytical column (Waters). Peptides were selected for fragmentation automatically by data-dependent analysis. Mass spectrometers were operated using Xcalibur software (version 4.1.31.9, *Thermo Scientific*).

**Table 1:** Sample demographics. Age, sex, level of operated intervertebral disc, and histological grade of degeneration in samples obtained from donors undergoing discectomy surgery for treatment of low back pain

Sample	Age (Years)	Sex	Operated Disc Level	Histology Grade	Severity
1	35	Female	L5/S1	4	Mild
2	44	Female	L5/S1	5	Mild
3	46	Female	L5/S1	6	Mild
4	55	Female	L5/S1	6	Mild
5	48	Male	L4/5	6	Mild
6	35	Female	L4/5	6	Mild
7	41	Female	L4/5	6	Mild
8	61	Male	L4/5	6	Mild
9	46	Male	L4/5	6	Mild
10	39	Male	L4/5	7	Mild
11	41	Female	L4/5	7	Mild
12	45	Male	L5/S1	7	Mild
13	29	Male	L5/S1	7	Mild
14	31	Male	L4/5	7	Mild
15	36	Female	L4/5	7	Mild
16	84	Female	L5/S1	7	Mild
17	35	Female	L4/5	7	Mild
18	30	Male	L5/S1	7	Mild
19	41	Female	L4/5	10	Severe
20	35	Male	L5/S1	10	Severe
21	40	Female	L5/S1	10	Severe
22	44	Female	L4/5	10	Severe
23	39	Male	L5/S1	10	Severe
24	51	Female	L5/S1	10	Severe
25	35	Male	L5/S1	10	Severe
26	31	Male	L4/5	10	Severe
27	42	Male	L4/5	10	Severe
28	42	Male	L5/S1	10	Severe
29	39	Male	L4/5	11	Severe
30	28	Male	L5/S1	11	Severe
31	49	Male	L5/S1	11	Severe
32	42	Male	L4/5	11	Severe
33	35	Female	L4/5	11	Severe
34	38	Female	L4/5	11	Severe
35	35	Female	L5/S1	11	Severe

### Tissue mass spectrometry: data analysis

Raw IVD tissue mass spectrometry data files (.raw) were imported into MaxQuant v2.3.1 for analysis [17, 18]. Raw spectra were searched against a reviewed human protein database (UniProt UP000005640, 20,420 entries, July 2024) in Andromeda, MaxQuant’s built-in database search engine. Label-free quantification (LFQ) was activated to quantify protein abundance across samples. Normalisation of intensities across samples was performed using the MaxLFQ algorithm within the MaxQuant software as described in Cox et al. [19]. Trypsin was selected as the digestion enzyme allowing a maximum of two missed cleavage sites. Oxidation of methionine and N-terminal acetylations were set as variable modifications, while carbamidomethylation of cysteine was set as a fixed modification allowing a maximum of five modifications per peptide. The first peptide search mass tolerance was set to 20ppm and the main search mass tolerance was set to 4.5ppm. Match between runs was disabled and the false discovery rate was set to 1% for peptides and proteins. All other parameters were left unchanged. MaxQuant output files were examined and the proteinGroups.txt file was used for downstream analysis.

LFQ intensities were imported into Perseus 2.0.11 for further analysis. Data were filtered to remove potential contaminants, proteins only identified by modification site, and reverse hits. Data was filtered so that proteins with valid LFQ intensities in 50% of all samples were kept, leaving a total of 119 proteins. Protein LFQ intensities for the 119 proteins were log_2_ transformed, and imputation was performed by replacing values with random numbers drawn from a normal distribution (width = 0.3, downshift = 1.8). Differential expression analysis between severe and mild degenerate IVD tissues was performed using a two-sample T-test and a permutation-based false discovery rate method was used for multiple comparisons. Data matrices were exported from Perseus for further analysis in R v4.2.2. and RStudio.

Principal component analysis was performed using the ‘*prcomp’* function in R and visualised using the ‘*autoplot’* function in ggplot2 [20]. Differentially expressed proteins were visualised using the EnchancedVolcano package [21]. KEGG enrichment pathway and gene ontology analyses were performed using Enrichr [22]. Gene set enrichment analysis was performed using clusterProfiler and visualised using enrichplot [23, 24]. Matrisome and non-matrisome proteins were categorised using the Naba et al. human matrisome database [25].

### Plasma mass spectrometry: protein extraction and SWATH-MS

To obtain plasma, whole blood was collected from the same donors as tissues at time of surgery. Whole blood was drawn into 9 ml red cap S-monovettes containing ethylenediaminetetraacetate tripotassium (K3-EDTA, *Sarstedt*). Within 30 min of collection, samples were centrifuged at 1500xg for 15 min at 4 °C to remove blood cells. Plasma supernatant was transferred to clean centrifuge tubes and spun at 2000xg for 14 min at 4 °C to remove any remaining cells. Plasma was then transferred to clean cryovials and frozen until use. For SWATH mass spectrometry (MS) analyses 10 μl of plasma was immunodepleted using Pierce Top 12 Abundant Protein Depletion Spin Columns (*Thermo Scientific*) following the manufacturer’s instructions. Depleted plasma was then concentrated, and buffer was exchanged by using Amicon Ultra-0.5 Centrifugal Filter Devices (*Merck-Millipore*).​ Total protein concentration was determined using a BCA protein assay kit (*Thermo Fisher*). The depleted plasma (containing 40 μg of protein) was denatured, reduced and alkylated in 25mM ammonium bicarbonate containing 5mM dithiothreitol (*GE Healthcare*), 50 mM iodoacetamide (*Sigma Aldrich*) and 1% sodium deoxycholate (Sigma Aldrich). Modified sequencing-grade trypsin (*Promega*) was added at a ratio of 10:1 substrate: enzyme and digestion was performed overnight at 37 °C. Digests were subsequently dried in a vacuum centrifuge GenevacTM (*Thermo Fisher Scientific*).​ Samples were reconstituted in loading buffer containing 2% (v/v) acetonitrile (*Thermos Fisher Scientific*), 0.1% (v/v) formic acid (*Thermo Fisher Scientific*), 100 fmol/μl PepCalMix (*MS Synthetic Peptide Calibration Kit*,* AB Sciex UK Ltd*) and 10 × index retention time (iRT) standards (*Biognosys AG*,* Switzerland*). Samples were analysed by SWATH-MS with a micro-flow LC-MS system comprising an Eksigent nanoLC 400 autosampler and an Eksigent nanoLC 425 pump coupled to an AB Sciex 6600 Triple-TOF mass spectrometer with a DuoSpray Ion Source. ​ Liquid chromatography gradient details and MS settings were as described by McGurk et al. [26].

### Plasma mass spectrometry: data analysis

Raw (.wiff) files were processed using DIANN software v1.8.1 [27]. First, for library-free search, an in silico-predicted spectral library was generated using the UniProt human proteome sequence database (UniProt Proteome ID: UP000005640, count: 20,654, July 2024). The generated spectral library was reuploaded into DIANN, and raw data was analysed using the robust LC (high precision) quantification strategy. Cross-run normalisation was set to ‘retention time-dependent’, and match between runs was disabled. The precursor false-discovery rate (FDR) was set to 1%. Recommended or default settings were used for all other parameters. The protein group output matrix and associated experiment annotation files were imported into FragPipe-Analyst software for further analysis [28]. Before differential expression analysis, the minimum percentage of non-missing values was set to 50% for all samples. No further normalisation was performed at this stage. Perseus-type imputation was performed for the remaining 277 proteins as described above. Differential expression was performed using Limma and the Benjamin Hochberg correction was used for multiple comparisons [29]. Data matrices were exported and visualised in R as described above.

### Immunohistochemistry

For immunohistochemistry, slides were deparaffinised and rehydrated as described above. Antigen retrieval was performed using citrate buffer pH 6 for 20 minutes at 95°C. Slides were allowed to cool to RT and endogenous peroxidase was blocked using 3% (v/v) hydrogen peroxide in industrial methylated spirit (IMS). Tissues were washed with tris-buffered saline (TBS) and non-specific binding was blocked using 25% (v/v) normal goat serum and 1% (w/v) bovine serum albumin (BSA) in TBS for 30 minutes at RT. Tissues were then incubated with 100μl of rabbit anti-human AEBP1 primary antibody (1/150, *Abcam AB254973*) overnight at 4°C. Slides were washed in TBS-Tween and incubated with 100μl of secondary goat anti-rabbit antibody in TBS (1/300) for 30 minutes at RT. Signal amplification was achieved by applying avidin/biotin complex solution (*Vector Laboratories*) to sections for 30 minutes at RT. Sections were rinsed in TBS and 3,3’-diaminobenzidine tetrahydrochloride (DAB) was used for signal detection. Excess DAB was tapped off and the sections were rinsed in deionised water. The slides were then counterstained using Mayer’s haematoxylin (5 min) and rinsed in tap water before they were dehydrated, cleared, and mounted with coverslips. Slides were imaged using an automated 3D Histech Pannoramic P250 slide scanner and processed using SlideViewer software (3DHISTECH).

### Intervertebral disc primary cell extraction and culture

Fresh IVD tissue was finely chopped and placed into a 50 ml tube containing 10 ml serum-free DMEM supplemented with 0.1% (w/v) collagenase type II (*Gibco*, *17101015*) and 2% (v/v) antibiotic-antimycotic solution (*Sigma Aldrich*). Tissues were incubated overnight at 37 °C. Cells were passed through a 70 μm cell strainer and centrifuged at 400xg for 5 min. The cell pellet was resuspended in 10 ml serum-free medium and centrifuged at 400xg for 5 min. Cells were resuspended in 5 ml complete disc cell media (high-glucose DMEM supplemented with 1mM sodium pyruvate, 10 μm L-ascorbic acid 2-phosphate sesquimagnesium salt hydrate, 1% antibiotic-antimycotic solution, and 10% fetal calf serum) and dispensed into T25 cell culture flasks. Cells were cultured at 37 °C and 5% CO_2_ until they reached 80% confluency (P0).

### RNA extraction, cDNA conversion, and quantitative reverse transcription polymerase chain reaction (qRT-PCR)

IVD primary cells were trypsinised and lysed using 1 ml TRI Reagent (*Sigma Aldrich*). Lysates were incubated at RT for 5 min. Chloroform (200 μl) was added and each sample was shaken vigorously before centrifugation at 12000xg for 20 min at 4 °C. 250 μl of the aqueous phase was transferred into a new tube containing 250 μl isopropanol. Samples were incubated at RT for 10 min and then centrifuged at 12000xg for 20 min at 4 °C to precipitate RNA. The supernatant was discarded, and RNA pellets were washed twice with ice-cold 70% ethanol and centrifuged at 8000xg for 5 min at 4 °C. RNA pellets were dried for 10 min at RT and eluted in 50 μl 1X Tris-EDTA solution. RNA concentration was quantified using a Nanodrop 1000 spectrophotometer and associated software (Thermo Fisher Scientific).

RNA (1 μg) from each sample was converted to cDNA using the High-Capacity RNA-to-cDNA Kit (*Applied Biosystems*) according to manufacturer instructions. cDNA was dilution to 5ng/μl and stored at -20 °C until use. For qRT-PCR, 1 μl of the diluted cDNA was mixed with 5ul Fast SYBR Green Master Mix (*Applied Biosystems*), 2.8 μl water and 0.2 μl of 10 μm human *AEBP1 (F: GAGAAGGAGGAGCTGAAGAAAC; R: CGGATCTGGTTGTCCTCAATAC) or GAPDH (F: GGTGTGAACCATGAGAAGTATGA; R: GAGTCCTTCCACGATACCAAAG)* forward and reverse primers (*Integrated DNA Technologies*). Data acquisition was performed on a StepOnePlus Real-Time PCR System and StepOne Software (*Applied Biosystems*).

### Statistics

Statistical tests were performed on Graph Prism Software v10. Unpaired t-tests were used to compare differences between severe and mild degenerate protein intensities and gene expression levels. Receiver operating characteristics (ROC) curve analysis was applied to proteins to evaluate the ability to distinguish between mild and severe IVD degeneration groups. The area under the curve (AUC) score was used to summarise the effectiveness of the selected proteins in differentiating mild and severe IVD degeneration, with proteins having an AUC score above 0.75 considered good discriminators. Correlational analysis was performed to evaluate relations between protein intensity and other parameters such as histology grade, and age. Statistical significance was set to *p* < 0.05 unless stated otherwise.

## Results

### Histological analyses of surgically excised intervertebral disc tissues reveal a spectrum of degenerative changes among donors undergoing discectomy for low back pain

**Fig. 1:**
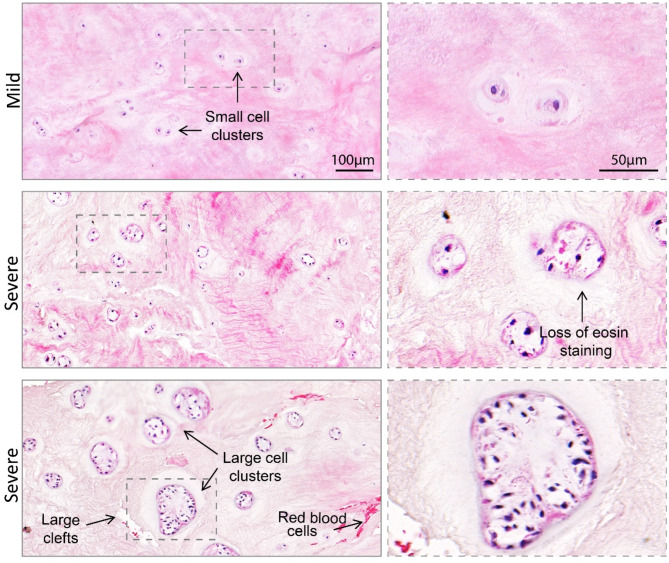
Histological analyses of surgically excised intervertebral disc tissues show varying degenerative changes among patients. Representative micrographs of graded haematoxylin and eosin-stained tissues showing histomorphological characteristics of mild and severe degenerate intervertebral disc tissues

Histological characterisation of IVD tissue excised during discectomy can provide insight into structural changes associated with the progression of degeneration not normally detected by other imaging techniques such as MRI. We examined the histopathological features of tissues from donors undergoing lumbar discectomy for LBP. Haematoxylin and eosin staining of these tissues revealed significant alterations in the morphological and cellular architecture of IVD tissue marked by the presence of known features of degeneration such as the formation of cell clusters, loss of eosin staining and the presence of fissures (Fig. 1) [5]. We also noted that the severity of degeneration was highly variable, with some individuals exhibiting more exacerbated degenerative features than others. Mildly degenerated IVD tissues had smaller cell clusters (2–3 cells), and narrow fissures, whereas severely degenerated tissues exhibited very large cell clusters (6 + cells), wider fissures, more pronounced loss of eosin staining and presence of red blood cells and small vessels indicative of neovascularisation (Fig. 1). These observations demonstrated that IVD tissues from donors undergoing surgery to treat LBP display varying degrees of microscopic degenerative changes that cannot be detected by standard diagnostic imaging techniques, highlighting the need for biomarkers that better reflect tissue changes occurring during IVD degeneration progression.

### Proteomic analyses show key differences in protein composition between mild and severe degenerated IVD tissues

**Fig. 2:**
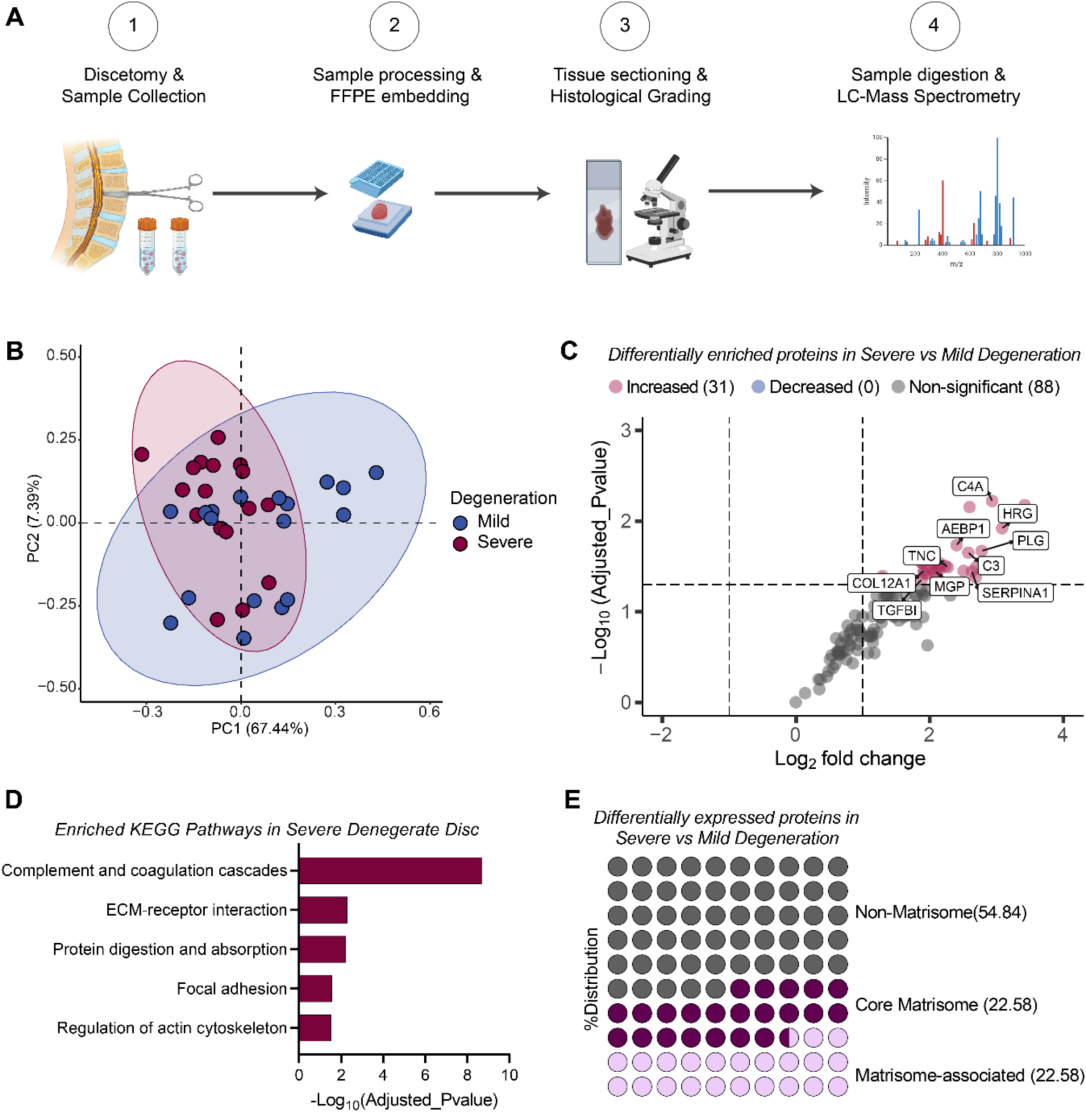
Discovery proteomic analyses reveal key changes in intervertebral disc (IVD) tissue protein composition associated with increased severity of degeneration. (**A**) Summary of workflow [1, 2]. Surgically excised disc tissues from patients treated for degenerative disc disorder were formalin-fixed and embedded in paraffin blocks [3]. Tissue sections were collected, stained, and blindly graded by an experienced histopathologist [4]. Tissues graded mild (*n* = 18) or severe (*n* = 17) were processed for analysis by liquid chromatography with tandem mass spectrometry (LC/MS/MS). (**B**) Unsupervised principal component analysis of tissue samples from mild and severe degenerate IVDs. (**C**) Differential enrichment analysis of protein levels between severe and mild degenerate IVD tissues. (**D**) Top five enriched KEGG pathways in severe degenerate IVDs in comparison to mild. (**E**) Plot illustrating proportions of significantly differentially expressed proteins classified into non-matrisome, core matrisome and matrisome-associated proteins

Histological assessment of degenerated IVD tissues revealed more widespread loss of eosin staining around cells in severely degenerated tissues compared to mild ones suggesting changes in ECM composition as degeneration progresses. To assess how the ECM composition changes in IVD tissues as degeneration progresses, we histologically classified excised tissues into two groups: mild degenerated (Grades 4–7, *n* = 18), and severe degenerated (Grades 10–12, *n* = 17) using the Sive et al. histological grading system [5]. Label-free mass spectrometry analysis was then applied to samples to compare protein abundance levels between the two groups (Fig. 2A). A median of 294 proteins (28 proteins unique to the group) was detected in the severe degenerated tissues, while a median of 270 proteins (4 exclusively present in the group) was detected in the mild degenerated sample group. Following quality control and filtration based on 50% valid values, 119 proteins were detected in both mild and severe degenerated sample groups. No proteins were uniquely present in each group. Unsupervised dimensionality reduction with principal component analysis showed distinct clustering between mild and severe degenerated samples suggesting differences in the proteomes of the two groups (Fig. 2B). Differential expression analysis revealed that the abundance of 31 proteins (adjusted_p < 0.05; Log_2_Foldchange (LFC) = ± 1) was significantly increased in severe degenerated samples compared to mild, including IGLC6, HRG, AEBP1, C4A, TNC and COL6A3 (Fig. 2C, Additional File 1). KEGG pathway enrichment analysis showed that the differentially distributed proteins were involved in complement system activation, ECM regulation, and protein digestion and absorption (Fig. 2D). Altogether, these results indicate that the severity of degeneration leads to changes in IVD protein composition.

To further elucidate changes in ECM protein composition in the IVD, we categorised the differentially expressed proteins between severe and mild degenerated IVD into non-matrisome, core matrisome and matrisome-associated proteins using the Naba et al. Matrisome Database [25]. Core matrisome and matrisome-associated proteins constituted 45.16% (14 proteins: 7 core matrisome proteins and 7 matrisome-associated proteins) while non-matrisome proteins accounted for 54.84% (17 proteins) of the differentially expressed proteins (Fig. 2E). Pathway enrichment analyses showed that enriched non-matrisome proteins were involved in antigen-receptor-mediated signalling, apoptotic cell clearance and phagocytosis (Supplementary Fig. 1A). Enriched matrisome-related biological pathways confirmed that differentially expressed matrisomal proteins were involved in ECM organisation, regulation of cell adhesion, collagen fibril organisation and axon guidance suggesting that biological processes associated with ECM remodelling and maintenance in the disc are further impaired as degeneration progresses (Supplementary Fig. 1B).

### AEBP1, TNC, MGP and, COL12A1 are the most increased core matrisome proteins as IVD degeneration worsens

**Fig. 3:**
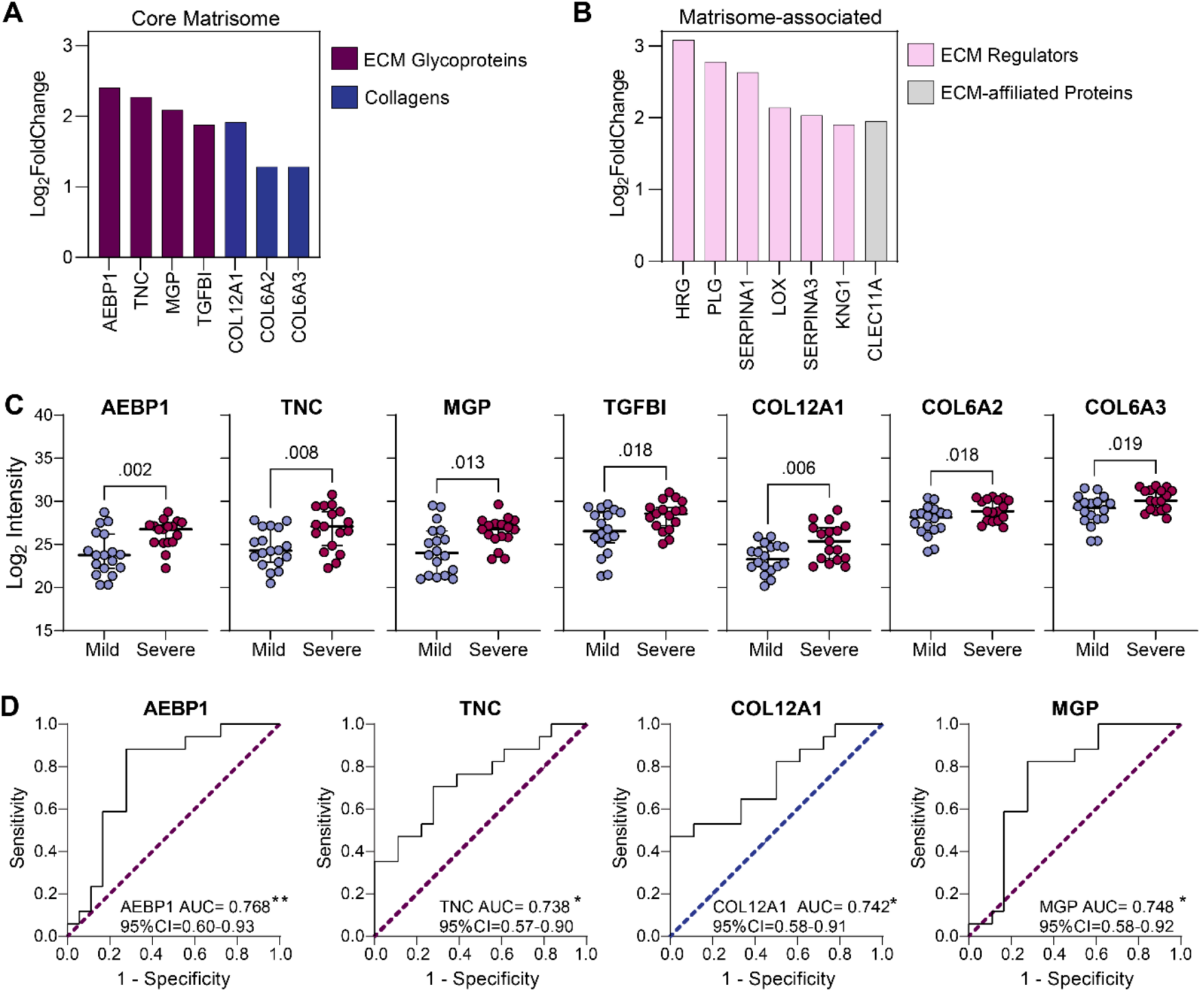
Core matrisome proteins AEBP1, COL12A1, TNC and MGP show the strongest relationship with the severity of intervertebral disc (IVD) degeneration. **A-B**. Bar chart showing log_2_ fold change of core matrisome (**A**) and matrisome-associated (**B**) proteins upregulated in severe compared to mild degenerate IVD tissues. **C**. Comparison of core matrisome protein mass spectrometry log_2_ intensities from mild (*n* = 18) and severe (*n* = 17) degenerate IVD tissues (unpaired t-test, data are shown as median with 95% confidence interval). **D**. Receiver operating characteristics curve analysis evaluating the accuracy of glycoproteins AEBP1, TNC and MGP, and collagen COL12A1, at distinguishing between mild (*n* = 18) and severe (*n* = 17) degenerate IVD tissues. The area under the curve score (AUC) and 95% confidence intervals are shown for each protein (Dotted line: random classifier; Sensitivity: true positive rate; 1-specificity: false positive rate; statistical significance: *p < 0.05, **p < 0.01)

Highly enriched core matrisome proteins in response to degeneration progression included ECM glycoproteins AEBP1, TNC, MGP, and TGFBI and collagens COL12A1, COL6A2, and COL6A3 (Fig. 3A). Among these, adipocyte enhancer binding protein 1 (AEBP1) was the most differentially enriched matrisome protein in severe compared to mild degenerated tissues (LFC = 2.40). In addition to AEBP1, other proteins such as tenascin C (TNC), matrix gla protein (MGP) and collagen type XII alpha 1 chain (COL12A1), also showed a significant increase in abundance in severe compared to mild degenerated tissues. On the other hand, enriched matrisome-associated proteins included ECM regulators involved in fibrinolysis such as histidine-rich glycoprotein (HRG), plasminogen (PLG) and serpin family A member 1 (SERPINA1) indicative of impaired fibrinolytic activity as degeneration worsens (Fig. 3B, Supplementary Fig. 2A).

We next examined the relationship of all core matrisome protein abundances with IVD tissue degeneration grade and found that protein levels of AEBP1, TNC, MGP and COL12A1 were the most significantly different between mild and severe degenerate tissues (Fig. 3C). These observations highlighted the potential for these proteins as markers for IVD degeneration progression. To confirm this, we performed receiver operating characteristics curve analyses on core matrisome and matrisome-associated protein intensities (Fig. 3D, Supplementary Fig. 2A). From the core matrisome proteins, AEBP1 had the highest area under the curve (AUC) score of 0.768 indicating that it was the most accurate at distinguishing between mild and severe degenerate tissue in comparison to TNC (0.739), MGP (0.748) and COL12A1 (0.742). In summary, this data emphasizes changes in ECM-related proteins in the pathogenesis of IVD degeneration and highlights their potential as biomarkers for discriminating between severe and mild cases of IVD degeneration.

### AEBP1 is a tissue biomarker for degeneration progression in the intervertebral disc

**Fig. 4:**
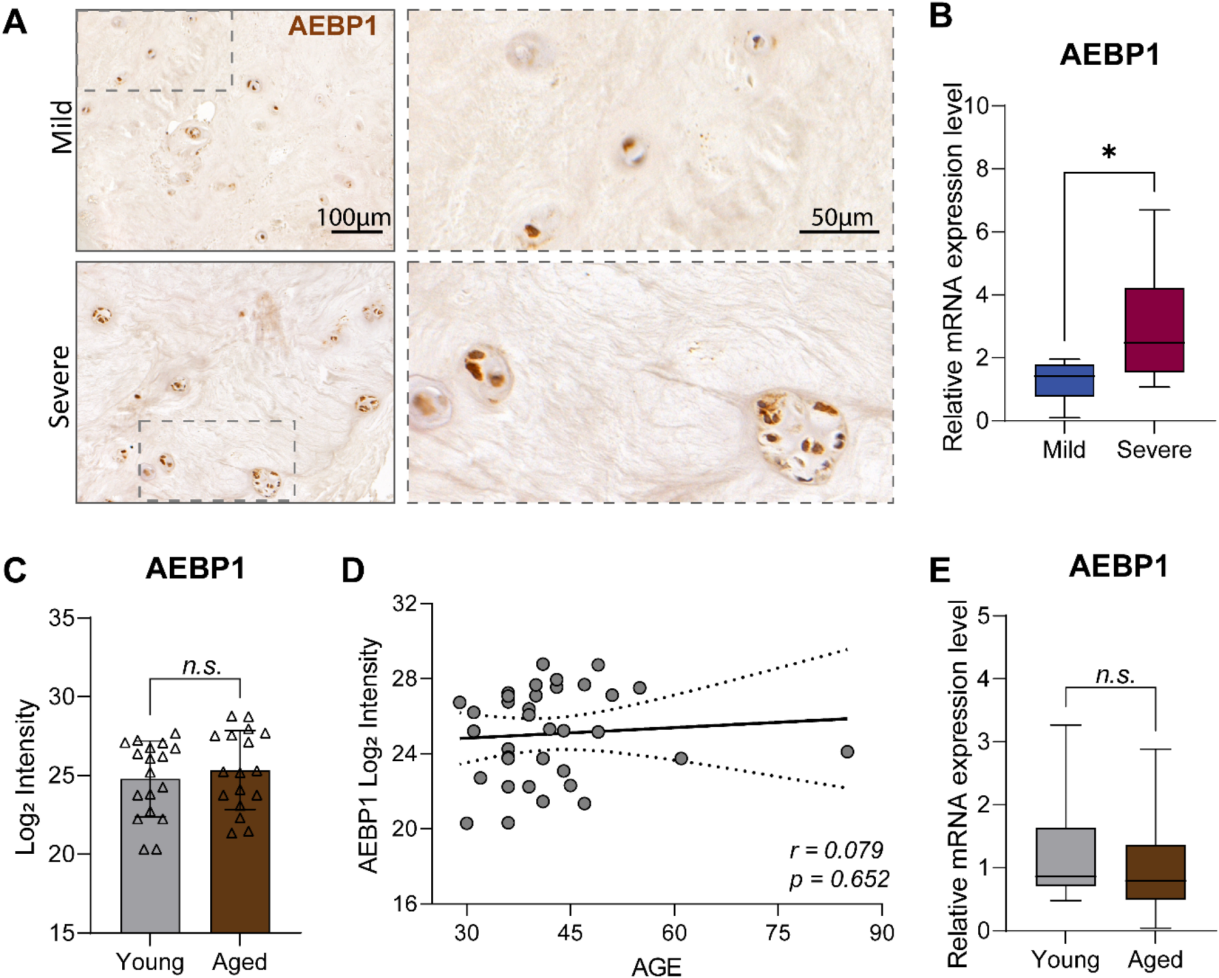
High levels of AEBP1 are associated with increased severity of intervertebral disc (IVD) degeneration but not ageing. (**A**) Representative micrographs of immunohistochemical staining of AEBP1 in mild vs. severe degenerate IVD tissues. (**B**) qRT-PCR analysis of AEBP1 gene expression in cells isolated from mild and severe degenerate IVD tissues. Data are shown as relative expression normalised to GAPDH (*n* = 10 per group, unpaired t-test, *p < 0.05). (**C**) Comparison of AEBP1 protein mass spectrometry log_2_ intensities from young (≤ 40 years old, *n* = 17) and aged (> 40 years old, *n* = 18) degenerated IVD tissues (unpaired t-test, *n.s*. = not significant, data are shown mean ± SD). (**D**) Correlation analysis between AEBP1 protein mass spectrometry log_2_ intensity and patient age (r = correlation coefficient). (**E**) qRT-PCR analysis of AEBP1 gene expression in cells isolated from young (≤ 40 years old, *n* = 6) and aged (> 40 years old, *n* = 14) degenerated IVD tissues. Data are shown as relative expression normalised to GAPDH (unpaired t-test, *n.s*. = not significant)

As AEBP1 was the most significantly changed core matrisome protein with the highest AUC score, we further evaluated its effectiveness as a distinguishing marker between mild and severe IVD degeneration. Immunohistochemical staining revealed increased AEBP1 staining intensity in severe degenerate tissues in comparison to mild with the more extensive protein expression observed within the large degeneration-association cell clusters in severe degenerate tissues (Fig. 4A). *AEBP1* RNA expression levels were also significantly increased in primary cells from severe degenerate IVD tissues in comparison to mild confirming that AEBP1 is altered at both protein and gene expression level (Fig. 4B). Interestingly, AEBP1 protein or gene expression levels showed no association with age (Fig. 4C-E), suggesting that changes in AEBP1 in the IVD are associated with the severity of degeneration and not influenced by age, thus AEBP1 could potentially serve as a marker for mild versus severe degeneration progression regardless of age.

### High levels of complement system proteins are associated with increased severity of IVD degeneration

**Fig. 5:**
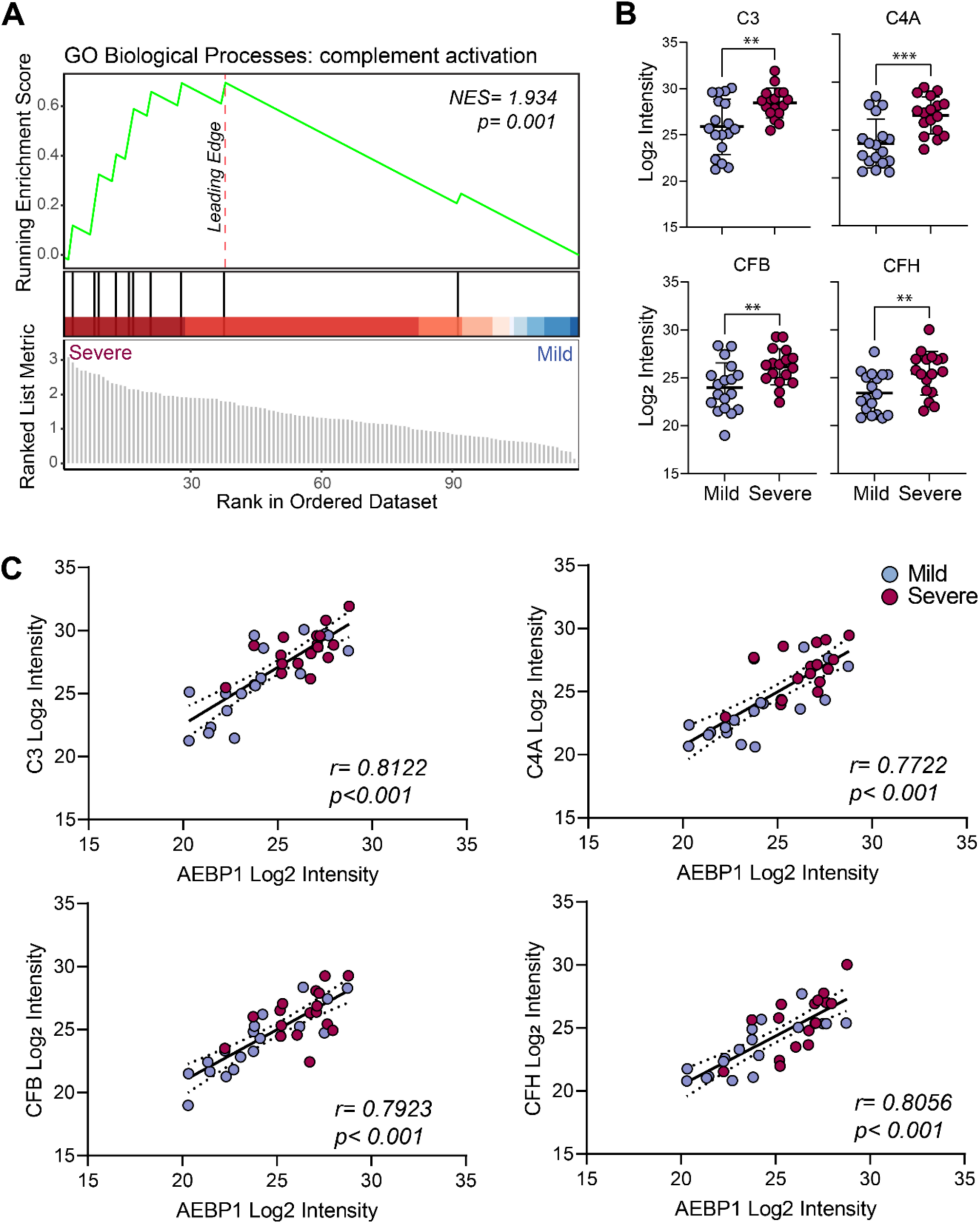
Complement activation pathway proteins are positively associated with the progression of intervertebral disc degeneration (IVD). (**A**) GSEA plot showing enrichment of the complement activation gene ontology biological pathway in severe vs. mild degenerate IVD tissue (*NES: normalised enrichment score*, *p* = *p*-value). (**B**) Comparison of C3, C4A, CFB, or CFH mass spectrometry protein log_2_ intensities from mild (*n* = 18) and severe (*n* = 17) degenerate IVD tissues (unpaired t-test, **p < 0.01, ***p < 0.001, data are shown as mean ± SD). (**C**) Correlation analyses between C3, C4A, CFB or CFH and AEBP1 mass spectrometry protein

Degeneration-associated changes in IVD tissues such as matrix degradation and cell apoptosis have been previously described to activate the complement system resulting in increased inflammation, neovascularisation and further tissue damage [30]. In this study, we found that complement system-related proteins were highly enriched in severe versus mild IVD tissues (Figs. 2D and 5A). The classical complement pathway component, C4A, was markedly increased in severe IVD tissues in comparison to mild. Similarly, alternative pathway proteins C3, CFH, and CFB were also significantly upregulated in severe compared to mild degenerated IVD (Fig. 5B). Strong positive correlations with AEBP1 were observed for all components indicating an association between AEBP1 levels, complement system activation and IVD degeneration progression (Fig. 5C). In summary, our data suggests that changes in both matrisome and non-matrisome related proteins in the IVD can be used simultaneously to monitor IVD disease progression.

### Plasma levels of A2M, F13B, MMP2, and IGF1 are correlated with histological grades of IVD degeneration

**Fig. 6:**
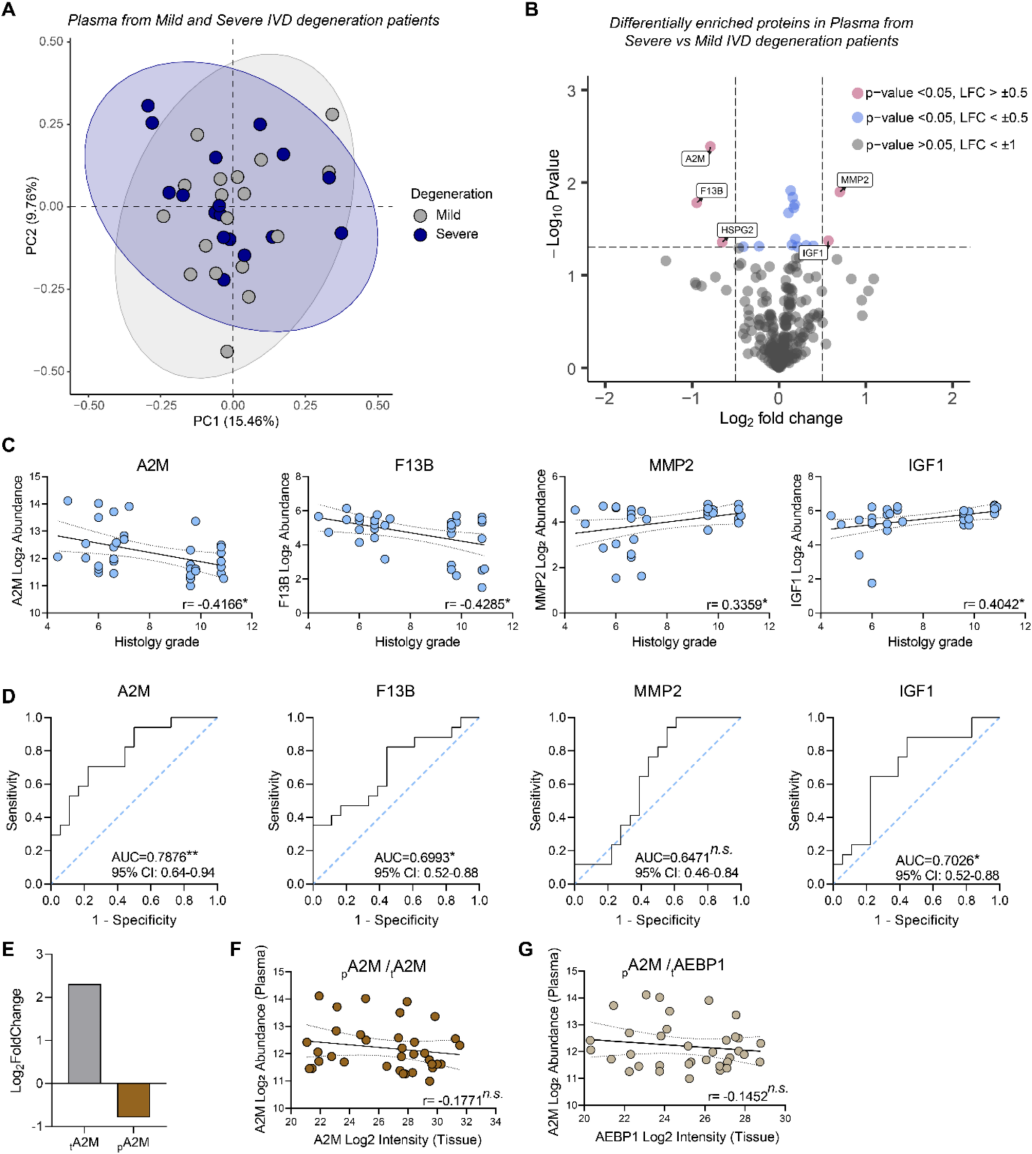
Associated plasma profiles from the same donors show a weak relationship with intervertebral disc (IVD) degeneration severity. **A**. Unsupervised principal component analysis (PCA) of associated plasma samples from the same donors with mild (*n* = 18) and severe (*n* = 17) IVD degeneration. **B**. Differential expression analysis of plasma proteins from donors with severe and mild IVD degeneration. **C**. Correlation analyses between plasma A2M, F13B, MMP2, or IGF1 and IVD tissue histology grade (*n* = 35, *n*.s.-not significant, *p < 0.05, r = correlation coefficient). **D**. Receiver operating characteristic curve analysis displaying the ability of plasma protein A2M, F13B, MMP2, or IGF1 to discriminate between donors with mild and severe IVD degeneration. The area under the curve score (AUC) is shown for each protein (Dotted line: random classifier; Sensitivity: true positive rate; 1-specificity: false positive rate; statistical significance: *p < 0.05, **p < 0.01, 95% confidence intervals are also shown). **E**. Bar charts comparing changes between tissue (t) and plasma (p) A2M log2foldchange. **F-G**. Correlation analyses between plasma A2M log_2_ abundance and tissue A2M (**F**) and tissue AEBP1 (**H**) protein log_2_ intensities (*n* = 35, *n*.s.-not significant, r = correlation coefficient)

While the identified tissue biomarkers offer a high correlation with degeneration severity, IVD tissue is not easily accessible and can only be obtained through highly invasive surgical techniques. Thus, to identify biomarkers that distinguish between mild and severe IVD degeneration and are also accessible with minimally invasive techniques, we performed mass spectrometry analyses on 35 blood plasma samples collected from the same individuals as the IVD tissues. Principal component analysis showed no distinct clustering between plasma samples from individuals with mild and severe degeneration plasma samples with both PC1 and PC2 explaining only 25.2% of the variance (Fig. 6A). Following data filtering based on 50% valid values, 277 proteins were detected in both groups. Differential expression analyses were applied to these to identify systemic changes as degeneration progressed. Only 5 proteins, A2M, F13B, HSPG2, MMP2, and IGF1, had a log_2_ fold change above 0.5 and a significance level less than 0.05 in severe compared to mild samples (Fig. 6B, Additional File 2). 21 of the 31 proteins differentially expressed in IVD tissue were also detected in plasma but no significant changes were observed in plasma for these proteins (Supplementary Table 1). F13B (coagulation factor XIII B chain), a zymogen involved in blood coagulation, was the most decreased protein plasma from donors with severe degeneration in comparison to mild (LFC= -0.95). A2M (alpha-2-macroglobulin), a protease inhibitor, was also reduced in donors with severe degeneration (LFC= -0.79). Conversely, MMP2 (matrix metalloproteinase-2), a zinc-dependent endopeptidase, was the most enriched (LFC = 0.70) followed by IGF1 (insulin-like growth factor 1) (LFC = 0.56) (Fig. 6B).

To investigate the potential use of A2M, F13B, MMP2, and IGF1 as biomarkers for IVD degeneration progression, we performed correlation analyses between these proteins and histological grades of degeneration. A2M and F13B had a moderate negative correlation with histological grade while MMP2 and IGF1 showed a moderate positive correlation with histological grade (Fig. 6C). ROC curve analyses revealed that A2M has the highest AUC score of 0.79, demonstrating its high accuracy in distinguishing between mild and severe IVD degeneration in donors when compared to F13B, MMP2 and IGF1 (Fig. 6D). This suggested that A2M could be a potential plasma biomarker for IVD degeneration progression.

### Plasma levels of A2M show weak correlations with proteins that are altered in IVD tissue as degeneration progresses

To determine if changes in plasma levels of A2M were related to those observed in the IVD tissue, we initially checked whether A2M was also present in our tissue data. We found that tissue A2M levels were increased in response to degeneration severity, showing an opposite trend to the one in plasma (Fig. 6E). Tissue A2M log_2_ intensities also exhibited a weak correlation with plasma A2M log_2_ abundancies indicating a poor relationship between the two (Fig. 6F). Additionally, we found that plasma A2M had no significant correlations with the 31 differentially distributed proteins in IVD tissue, including core matrisome AEBP1 (Fig. 6G, Additional File 2). In summary, these results suggest a poor alignment between plasma and tissue protein changes in donors with mild and severe disc degeneration. Given the complexity of systemic plasma changes, a larger sample cohort may be required to improve the identification of plasma proteins related to the progression of IVD degeneration.

## Discussion

Several studies have assessed changes in the proteome and transcriptome of non-degenerate and degenerated IVD tissues from adult humans [31–35]; however, investigations aimed at characterising pathophysiological changes at different stages of degeneration progression and how these affect ECM as well as blood protein composition have been limited. In this study, we histologically analysed and graded IVD tissue from donors undergoing discectomy surgery for the treatment of low back pain and found that some of the tissues exhibited more severe histological features of disc degeneration [5]. More severely degenerated tissues exhibited larger cell clusters and extensive loss of pericellular ECM compared to mild degenerated tissues. Increased number and size of cell clusters in the IVD are a hallmark of advancing disc degeneration in humans [36–38]. Moreover, cell clusters were found to be associated with increased MMP1 and loss of proteoglycans in severely degenerated discs in comparison to mild degenerated discs suggesting that changes in the cellular landscape of the IVD may influence ECM composition as degeneration progresses [38]. Despite this, differences in ECM protein composition in mild and severe degenerated tissues have not been fully defined.

We characterised the matrisome profile of severe degenerated IVDs in comparison to mild to identify ECM proteins associated with degeneration progression. We observed a shift to a more profibrotic matrix environment in severe degenerated IVD tissues characterised by high levels of collagens, COL12A1, COL6A2, and COL6A3 as well as ECM glycoproteins, AEBP1, TNC, MGP, and TGFBI, all which are associated with increased fibrosis in the IVD and other tissues [39–42]. Our results showed that AEBP1, TNC, MGP, and COL12A1 were the most increased core matrisome proteins as degeneration progressed. We thus evaluated the potential of these 4 proteins to be used as tissue biomarkers for distinguishing progressing stages of degeneration. ROC curve analyses showed that AEBP1, TNC, MGP and COL12A1 accurately differentiated between mild and severe degenerated IVD tissues, highlighting the potential of these proteins as a panel of biomarkers for degeneration progression.

AEBP1 was the most differentially distributed ECM protein between mild and severe degenerated IVD tissues, exhibiting the highest sensitivity and specificity as a biomarker for degeneration progression. AEBP1 is a protein encoded by the gene *AEBP1*, existing in two functionally distinct isoforms (*Q8IUX7-1* and *Q8IUX7-2*, UniProt, release 2024_04). AEBP1 isoform 1, also known as aortic carboxypeptidase-like protein (ACLP), is described as a secreted 1158 amino acid long protein which consists of an N-terminal signalling peptide, a lysine-proline-serine rich region, a collagen-binding discoidin domain and a carboxypeptidase-like domain [43–45]. AEBP1 isoform 1 has been linked to the ECM and is increased during vascular smooth muscle cell differentiation [45]. In addition, AEBP1 isoform 1 is highly expressed in collagen-rich tissues such as the skin, blood vessels, liver, lung, and IVD [40, 45–48]. AEBP1 isoform 2 is a truncated version lacking the long N-terminal region in isoform 1. This isoform is described as a transcriptional repressor expressed in the nuclear region of adipocytes and osteoclasts [49, 50]. Our immunohistochemical analyses showed that AEBP1 staining was localised within the cytoplasm and in the pericellular and extracellular matrices but not in the nuclei suggesting that the isoform detected in this study is likely to be isoform 1 rather than isoform 2.

Changes in *AEBP1* RNA levels have been previously described in the disc. A recent study demonstrated increased *AEBP1* gene expression in Pfirmann-graded late IVD degeneration compared to early IVD degeneration in human tissues [51]. Similarly, we found that cells from severe degenerated IVD tissues expressed higher levels of *AEBP1* in comparison to mild. Altogether, these findings validate that AEBP1 is increased at both gene expression and protein levels as degeneration progresses. Increased levels of AEBP1 have been previously implicated in other degeneration-related conditions. Higher AEBP1 levels were found in the articular cartilage of donors with osteoarthritis in comparison to normal counterparts. AEBP1 knockdown in mouse models of osteoarthritis revealed that loss of AEBP1 reversed degeneration association inflammation and ECM degradation [52]. Another study found that ACLP/AEBP1 was elevated in human fibrotic lung tissue, with *AEBP1* knockout mice exhibiting fewer myofibroblasts and less collagen in the lung following bleomycin injury in comparison to wild-type mice [48]. Furthermore, *AEBP1* gene expression levels were increased in severe liver fibrosis compared to normal liver [40]. Conversely, loss of *AEBP1* in mice was found to be detrimental to wound healing progression where regulation of ECM organisation is key for proliferative and remodelling phases of healing [47]. In summary, these studies highlight that normal levels of AEBP1 are important in the maintenance of the ECM microenvironment and increased levels drive ECM degradation and fibrosis, both of which are known features of degenerating tissues including the IVD.

Another well-documented feature of IVD degeneration is increased neovascularisation [53–55]. AEBP1 has been demonstrated to have proangiogenic effects in several tissues. It is upregulated during vascular smooth muscle differentiation and was found to regulate vascular adventitial progenitor differentiation following injury [45, 56]. AEBP1 levels were also elevated in tumour endothelial cells thereby promoting angiogenesis within the tumour microenvironment. Downregulation of AEBP1 in tumour endothelial cells in vitro reduced levels of angiogenesis-related genes, *POSTN* and *AQP1*, suggesting that AEBP1 may regulate new blood vessel formation [57]. Based on these findings, AEBP1 may also promote neovascularisation during degenerative disc disease; however, further research is required to elucidate the specific role of AEBP1 in IVD neovascularisation and to assess its potential as a therapeutic target for degenerative disc disease. In addition to AEBP1, other matrisome-associated proteins previously shown to regulate neovascularisation in disease, such as PLG, HRG, LOX, SERPINA3 and, CLEC11A were also found in higher levels in severely degenerated tissues [58–62]. This data supports that blood vessel formation and remodelling may be further enhanced as degeneration progresses, although again further work is required to confirm the presence and association of these matrisome proteins with the vasculature or endothelial cells in the degenerated IVD tissues.

We also showed that high AEBP1 protein levels were strongly correlated with an increased abundance of complement system proteins, including, C4A, C3, CFB and CFH. While AEBP1 has not been previously shown to regulate the complement system, a study in glioblastoma tissues also found that high AEBP1 expression levels were associated with enrichment for complement and coagulation cascade pathway-related genes [63]. Similar to our findings, this observation suggested a possible direct or indirect association between AEBP1 and the complement system in disease. A few recent studies have also reported increased levels of complement system proteins and genes in degenerated IVD tissues [64, 65]. Complement pathway proteins are predominantly synthesised in the liver and circulate in the blood. The lack of alteration in complement pathway proteins in the plasma proteome in this study, therefore, suggests that elevated tissue complement proteins are due to increased deposition or retention of these proteins in the damaged IVD. However, it remains unclear whether this complement pathway protein deposition is a result of, or is a contributor to increased degeneration in the disc (or both) [30]. Complement pathway activation has been shown to regulate angiogenesis in pathology [66]. As such, it is possible that AEBP1 and complement components act in synergy to promote angiogenesis, thereby contributing to degeneration. On the other hand, the complement system is activated in response to tissue damage and plays a major role in the clearance of apoptotic cells [67, 68]. Apoptotic cells are increased in degenerated IVD tissues which could result in increased activation of the complement pathway as degeneration worsens [69]. However, deposited complement proteins also further recruit and activate other immune cells, including monocytes and macrophages which can release proteases and cause further damage to ECM-rich structures which may result in further activation of the complement pathway proteins through feedback regulation hence their high abundance as degeneration progresses [70].

Our data suggests that AEBP1 may act as a tissue biomarker for monitoring degeneration progression in the tissue. However, IVD tissue is not readily accessible and is often obtained through highly invasive post-surgical procedures meaning its efficacy as a diagnostic tool is limited. Proteins identified in more accessible specimens such as urine or blood would make better biomarkers for monitoring IVD degeneration progression. We integrated proteome data from matched tissue and plasma samples from donors with severe and mild IVD degeneration to identify associated plasma biomarkers. We found that protein levels of A2M, a protease inhibitor, were differentially distributed in the plasma of donors with severe IVD degeneration compared to mild. Previous studies have shown that alterations in A2M may affect IVD function. For example, reduced A2M levels were found in severe degenerated NP tissues expressing high levels of reactive oxygen species and the addition of exogenous A2M reduced levels of oxygen reactive species in cultured NP, suggesting that A2M plays an antioxidative role in the IVD [71]. A2M also inhibited inflammation and reduced expression of degradation enzymes in human chondrocytes, but increased levels of protective matrix genes such as aggrecan in vitro [72]. Furthermore, the injection of autologous A2M was found to alleviate discogenic back pain in humans [73]. These studies demonstrate that A2M may play a protective role in the IVD and alteration in levels may impair disc function. Therefore, it is possible that reduced plasma levels of A2M in donors with severe IVD degeneration may be linked to its pathogenesis and progression. Nonetheless, plasma A2M had weak associations with proteins altered in IVD tissues. This indicated that A2M could not be used as a sole plasma biomarker for IVD degeneration progression despite a moderate AUC score of 0.78. A2M can potentially be combined with other parameters, such as age, body mass index, tissue markers, or other routinely monitored blood markers, to improve its sensitivity in predicting degeneration progression. A recent study demonstrated that a combination of age, C-reactive protein and CCL22 plasma levels could efficiently predict the recovery of patients who underwent spine surgery to treat disc degeneration [74]. However, sensitivity was reduced when these markers were used individually implying that plasma biomarkers for IVD degeneration are more efficient when combined with other parameters. The weak relationship observed between plasma and tissue protein levels may also be due to small cohort sizes used here. A larger cohort size may be necessary to fully characterise changes in plasma levels of A2M in relation to disc degeneration, although a recent study which analysed 100 serum samples from subjects with and without modic changes found no correlation between serum protein levels and modic changes [75]. Altogether, these results suggest that much larger population studies are required to fully characterise the relationship between blood biomarkers and IVD degeneration.

## Conclusion

In summary, our data demonstrates that ECM protein composition is dysregulated as IVD degeneration worsens. We show elevated protein levels of AEBP1, a collagen-binding protein that has been previously implicated in increased fibrosis, angiogenesis and ECM degradation, in severe degenerate tissues in comparison to mild tissues. We further suggest that AEBP1 is a potential tissue biomarker for monitoring degeneration progression. However, histologically observed tissue changes in the disc need to be integrated with non-invasive methods of evaluating IVD degeneration such as blood biomarkers, to aid in the diagnosis, monitoring, prognosis and treatment of the disease.

## Electronic supplementary material

Below is the link to the electronic supplementary material.

Supplementary Material 1

Supplementary Material 2: **Title of the data**: Differentially distributed proteins from mass spectrometry analyses of severe and mild degenerated intervertebral disc tissues. **Description of the data**: List of Log2 LFQ protein intensities and differentially distributed proteins obtained from label-free mass spectrometry analyses of severe and mild degenerated intervertebral disc tissues.

Supplementary Material 3: **Title of the data**: Differentially distributed proteins from mass spectrometry analyses of plasma from donors with severe and mild intervertebral disc degeneration. **Description of the data**: 1. List of Log2 protein intensities and differentially distributed proteins obtained from DIA SWATH mass spectrometry analyses of plasma from donors with severe and mild intervertebral disc degeneration. 2. Correlation analyses between plasma A2M and proteins changed in IVD tissue in response to degeneration progression.

## Data Availability

The datasets generated and analysed during the current study have been deposited to the ProteomeXchange Consortium via the PRIDE - PRoteomics IDEntifications repository with identifiers “PXD055158” for plasma SWATH DIA data and “PXD056620” for IVD tissue LC-MS-MS data [76].

## Abbreviations

A2M: Alpha 2-macroglobulin
AEBP1: Adipocyte enhancer binding protein 1
AUC: Area under the curve
ECM: Extracellular matrix
IVD: Intervertebral disc
ROC: Receiver operating characteristic curve
