# Supplementary material for: Proteomic profiling of human plasma and intervertebral disc tissue reveals matrisomal, but not plasma, biomarkers of disc degeneration
Source: Arthritis Res Ther. 2025 Feb 10;27:28. doi: 10.1186/s13075-025-03489-9 (PMC11809052; doi:10.1186/s13075-025-03489-9)
Supplement: Supplementary file 1 — Supplementary Material 1 [file 13075_2025_3489_MOESM1_ESM.docx]

Supplementary Materials


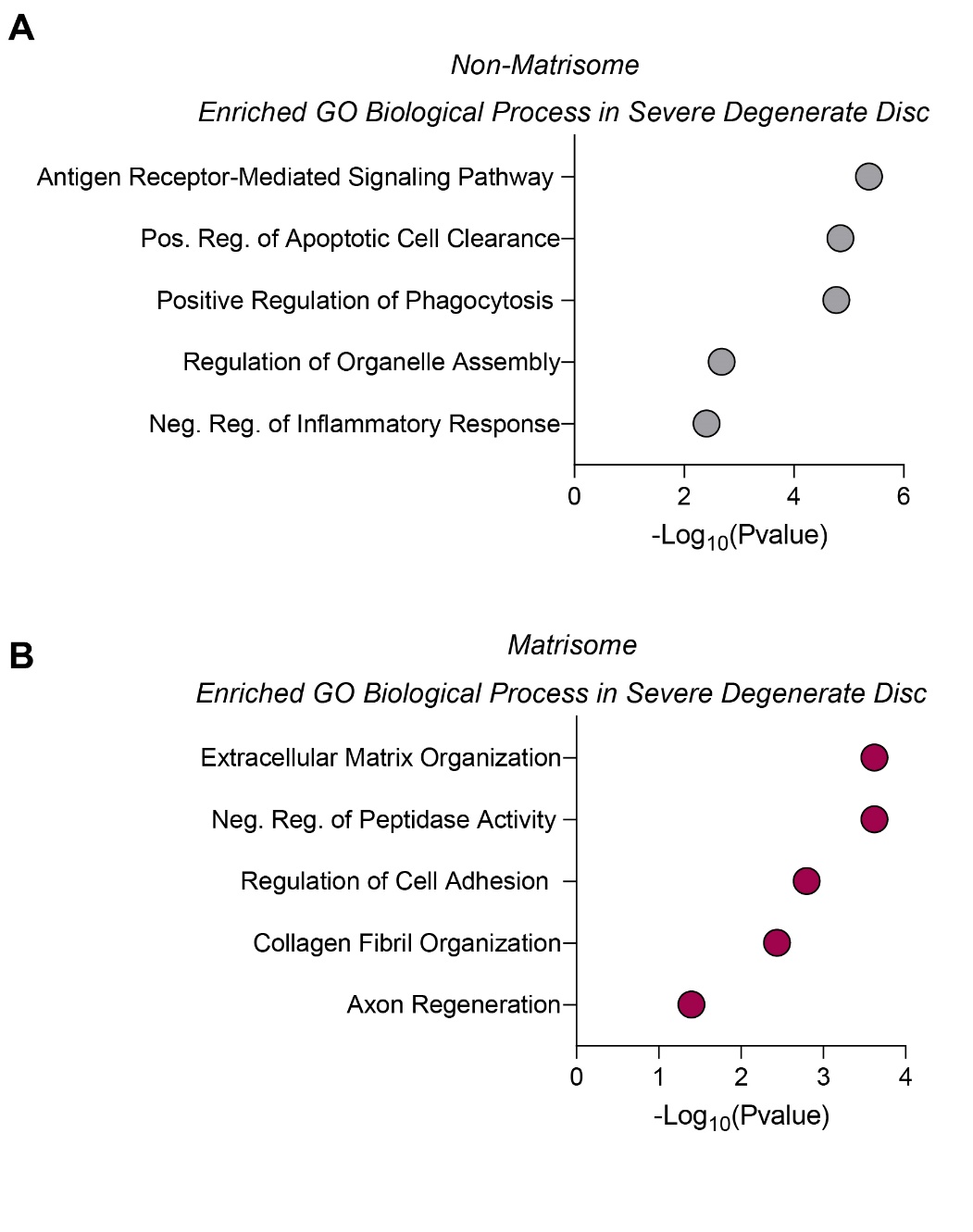
**Supplementary Figure 1:** Gene ontology (biological processes) pathway enrichment analysis for non-matrisomal (**A**) and matrisomal (**B**) proteins differentially expressed in the severe versus mild degenerate intervertebral disc.


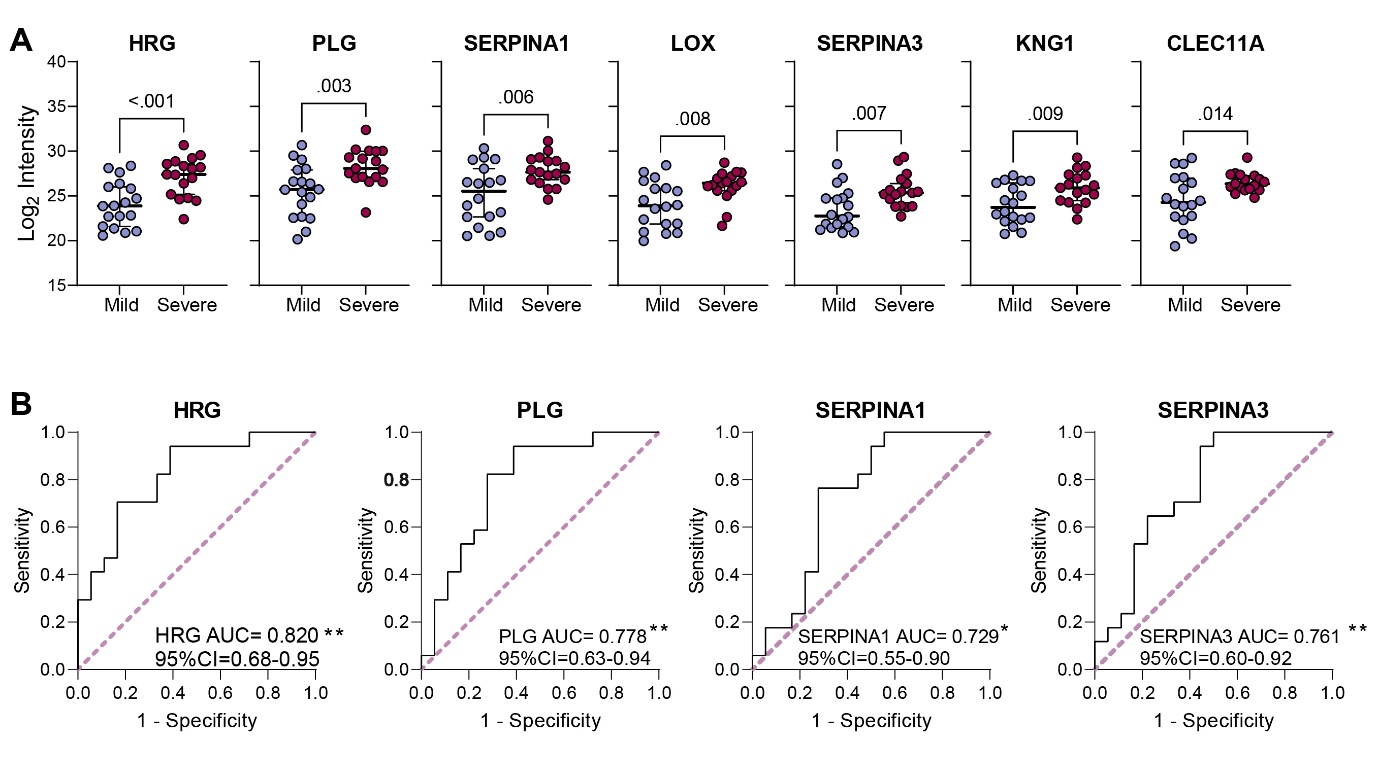


**Supplementary Figure 2**. **A.** Comparison of matrisome-associated protein mass spectrometry log_2_ intensities from mild (n=18) and severe (n=17) degenerate IVD tissues (unpaired t-test, data are shown as median with 95% confidence interval). **B.** Receiver operating characteristics curve analysis evaluating the accuracy of ECM regulators HRG, PLG, SERPIA1, and SERPINA3 at differentiating between mild (n=18) and severe (n=17) degenerate IVD tissues. The area under the curve score (AUC) and 95% confidence interval is shown for each protein (Dotted line: random classifier; Sensitivity: true positive rate; 1-specificity: false positive rate; statistical significance: *p< 0.05, **p<0.01).

**Supplementary Table 1** Comparison of fold changes between tissue and plasma from donors with severe and mild degeneration. List of log2 fold changes and p values for differentially expressed proteins in severe versus mild degenerate intervertebral disc (IVD). Changes observed in matched blood plasma are also shown for the same proteins.

|  | **IVD TISSUE** | | **PLASMA** | |
| --- | --- | --- | --- | --- |
|  | **Log2 fold change** | **p-value** | **Log2 fold change** | **p-value** |
| AEBP1 | 2.402 | 0.002 | - | - |
| APCS | 1.887 | 0.010 | -0.030 | 0.849 |
| APOA1 | 2.687 | 0.016 | 0.035 | 0.879 |
| C3 | 2.577 | 0.004 | 0.033 | 0.621 |
| C4A | 2.933 | 0.001 | 0.019 | 0.903 |
| CFB | 2.153 | 0.008 | -0.066 | 0.416 |
| CFH | 2.063 | 0.009 | -0.081 | 0.548 |
| CLEC11A | 1.954 | 0.014 | - | - |
| COL12A1 | 1.914 | 0.006 | - | - |
| COL6A2 | 1.286 | 0.018 | - | - |
| COL6A3 | 1.284 | 0.019 | -0.346 | 0.272 |
| GSN | 2.233 | 0.008 | 0.090 | 0.283 |
| HRG | 3.083 | 0.001 | 0.153 | 0.146 |
| IGHA1 | 2.596 | 0.001 | 0.493 | 0.149 |
| IGHG1 | 1.915 | 0.012 | 0.083 | 0.748 |
| IGHG3 | 2.146 | 0.007 | -0.118 | 0.842 |
| IGKC | 1.881 | 0.009 | 0.057 | 0.811 |
| IGLC6 | 3.422 | 0.001 | - | - |
| KNG1 | 1.905 | 0.009 | 0.111 | 0.085 |
| LOX | 2.139 | 0.008 | - | - |
| MGP | 2.087 | 0.013 | - | - |
| MSN | 2.055 | 0.009 | - | - |
| PGLYRP2 | 2.071 | 0.013 | -0.092 | 0.268 |
| PLG | 2.777 | 0.003 | -0.033 | 0.606 |
| SERPINA1 | 2.631 | 0.006 | -0.097 | 0.531 |
| SERPINA3 | 2.031 | 0.007 | 0.017 | 0.838 |
| TF | 2.695 | 0.006 | -0.264 | 0.105 |
| TGFBI | 1.881 | 0.018 | -0.022 | 0.821 |
| TNC | 2.270 | 0.008 | - | - |
| TTR | 2.506 | 0.013 | 0.107 | 0.281 |
| VIM | 1.294 | 0.015 | - | - |
